# Genome-wide polyadenylation site mapping datasets in the rice blast fungus *Magnaporthe oryzae*

**DOI:** 10.1038/sdata.2018.271

**Published:** 2018-11-27

**Authors:** Marco Marconi, Ane Sesma, Julio Luis Rodríguez-Romero, María Lourdes Rosano González, Mark D. Wilkinson

**Affiliations:** 1 Centro de Biotecnología y Genómica de Plantas UPM – INIA, Madrid, Spain; 2 Swiss Federal Institute of Technology, Zurich, Switzerland

**Keywords:** Gene regulation, RNA modification, Genome informatics, RNA sequencing

## Abstract

Polyadenylation plays an important role in gene regulation, thus affecting a wide variety of biological processes. In the rice blast fungus *Magnaporthe oryzae* the cleavage factor I protein Rpb35 is required for pre-mRNA polyadenylation and fungal virulence. Here we present the bioinformatic approach and output data related to a global survey of polyadenylation site usage in *M. oryzae* wild-type and Δ*rbp35* strains under a variety of nutrient conditions, some of which simulate the conditions experienced by the fungus during part of its infection cycle.

## Background & Summary

*Magnaporthe oryzae* is an economically relevant fungal pathogen of cereals, and represents an important threat to food security, particularly for cereal-dependent populations^1^. It is responsible for the devastating blast disease in rice and other grasses such as wheat, finger millet and maize^1^. The RNA-binding protein Rbp35 has been characterized in this fungus^2,3^. No clear orthologues of this protein are present in budding yeast. The Δ*rbp35* mutant shows reduced virulence on leaves and roots of rice. Interestingly, this protein interacts with CFI25, the homologue of human CFIm25^4,5^, indicating that Rbp35 is a component of the fungal polyadenylation machinery. In fact, Rbp35 is required for the alternative polyadenylation (APA) of pathogenicity-associated genes^3^.

The pre-mRNA 3′ end processing occurs in two steps. First, the pre-mRNA is cleaved and, subsequently, a polyadenosine (poly(A)) tail is added^6^. Unfortunately, standard high throughput RNA sequencing does not allow the identification of polyadenylation sites (PASs) since only a small percentage of sequencing reads contain poly(A) tails. To circumvent this problem, several techniques have been developed to enrich the transcript ends of poly(A)+RNAs (reviewed in Marconi *et al.*^7^).

Here, we present the datasets derived from a genome-wide PASs profiling within the *M. oryzae* wild type isolate Guy11 and the Δ*rbp35* mutant using the 3′T-fill method^8^. The datasets include three biological replicates in four nutritional conditions including nitrogen and carbon starvation. These data will help to identify the coordinated RNA networks that regulate *M. oryzae* growth in starved cells as well as gene networks regulated by Rbp35. These data also provide useful information for enhancing genome annotations and for cross-species comparisons of PASs and PAS usage within the fungal kingdom. The biological analyses of these data have been published^9^.

## Methods

### Fungal media, growth conditions and infection assays

*M.oryzae* strain Guy11 (WT) and 2D4 (Δ*rbp35*) were grown on CM (complete medium), MM (minimal medium) and DCM (defined complex medium)^10^. Starvation conditions were carried out on MM depleted of nitrogen or carbon sources.

### RNA extraction, library preparation and RNA sequencing

Fungal material was harvested from 60 hour-old fungal mycelia grown in liquid CM (24 h) and then transferred for an additional 12 h in a fresh CM, MM, MM-N or MM-C. Three biological replicates were independently harvested and extracted for each library preparation. Total fungal RNA was isolated by a standard trizol method^11^ and treated with RNase-free DNaseI using Turbo DNA-free kit (Ambion). Library preparation was carried out as described by the EMBL (Heidelberg) sequencing service, and similarly, the sequencing libraries and the 3’T-fill reaction were prepared as previously described^8^. The final libraries were loaded into the cluster station (cBot, Illumina) and the priming buffer was exchanged for the T-fill solution: 101 µl water, 20 µl Phusion buffer HF (5 ×) (NEB), 3 µl dTTPs (10 mM), 0.8 µl genomic DNA Sequencing primer V2 (100 µM) (Illumina), 3 µl non-hot start Phusion polymerase (2 U/µl, NEB) and 2 µl Taq polymerase E (5 U/µl; Genaxxon). The addition of the latter Taq polymerase was crucial for a complete T-fill. After clustering, the samples were sequenced on a HiSeq 2000 (Illumina).

### RNA sequencing and PAS detection protocol

**Figure 1:**
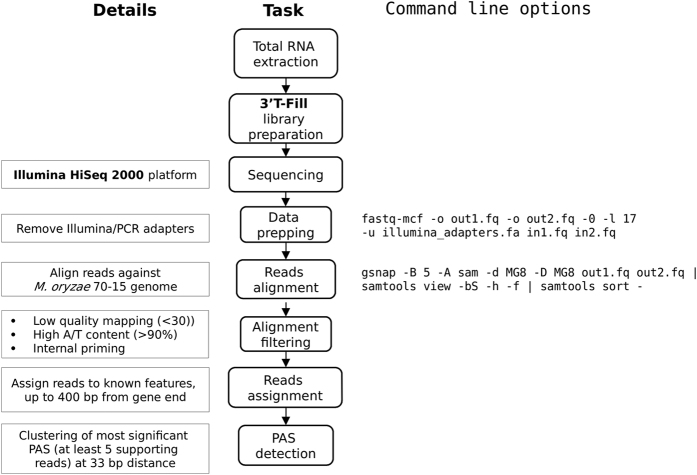
Workflow describing the steps followed to analyze 3′T-fill sequencing data. After total RNA extraction, Illumina RNA libraries were created following the specifics of the 3′T-Fill protocol^8^. RNA sequencing was then performed on the Illumina HiSeq2000 platform. FastQ reads were processed by removing sequencing adapters and finally aligned onto the reference genome. Alignments were further processed, filtering out low quality mappings, mappings with high A/T content and potential internal priming. Reads were thereafter assigned to overlapping genes up to 400 bp from the annotated gene end. Finally, identified poly(A) sites were clustered together in order to select high-confidence poly(A) sites.

**Figure 2:**
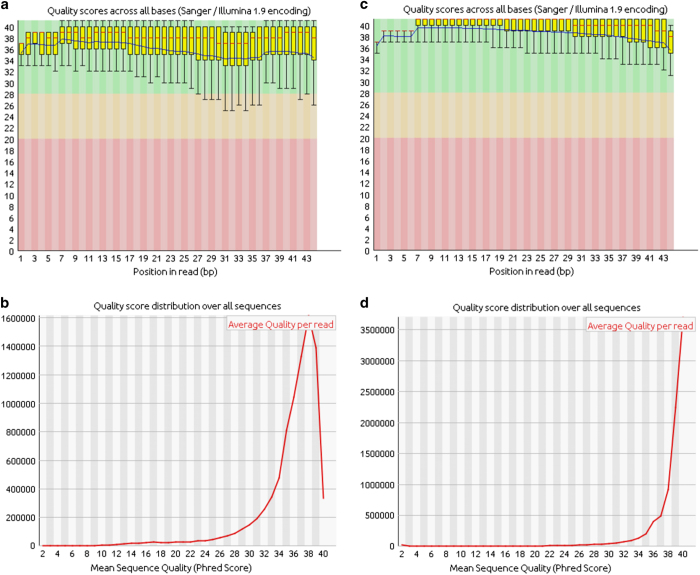
RNA sequencing quality details. FastQC report illustrating the average quality scores across all bases of the paired-end datasets for the sample wild-type/complete medium/replicate 1 (SRR6289246). (**a**,**c**) Phred quality scores for each nucleotide position are represented as a box and whisker plot. The central red line is the median value. The yellow box represents the inter-quartile range (25–75%). The upper and lower whiskers represent the 10 and 90% points. The blue line represents the mean quality. (**b**,**d**) Average quality per read along the read.

**Figure 3:**
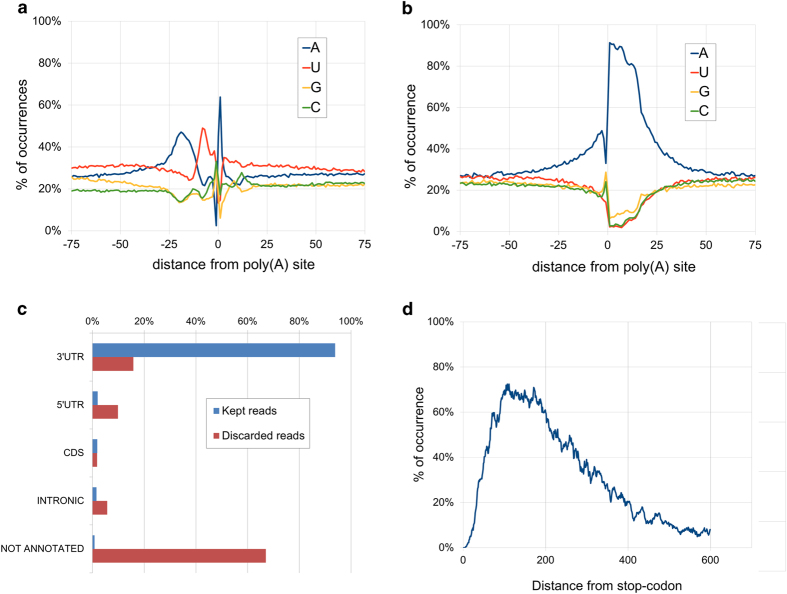
Details about the selection criterion for filtering and assignment of RNA sequencing read alignments. **(a)** Nucleotide profile of final high-confidence PASs. (**b**) The nucleotide profile around an alignment resulting from products of internal priming. (**c**) Comparison between mapped location of discarded reads (low quality, high A/T content and potential internal primings) and final kept reads. (**d**) Distance distribution of aligned reads from the closest annotated stop-codon. Reads rarely mapped greater than 400 bp from the closest annotated stop-codon, indicating that this was the typical 3’UTR length of *M. oryzae* mRNAs.

Fig. 1 provides a schematic of the workflow leading to PAS calling. Quality evaluation of sequence reads was performed with FastQC^12^, with the results shown in Fig. 2. De-multiplexed samples were trimmed to remove poly(A) tails and Illumina adapter sequences using the fastq-mcf utility from the ea-utils package (https://expressionanalysis.github.io/ea-utils/), keeping only reads with at least 17 nucleotides after trimming. Read-mapping was performed using STAR^13^ with default parameters. The alignments obtained were later filtered removing low-quality mapping (MAPQ < 30, see https://samtools.github.io/hts-specs/SAMv1.pdf), and mappings with high A/T content ( >80%) and potential internal primings (more than seven consecutive As in the first 5’ nucleotides or more than twelve As in the the first sixteen nucleotides, which is the length of the oligodT primers used during the 3′T-fill protocol); these alignments were discarded because they are suspected to be potential internal primings with genomic regions rich in As. In fact, a closer analysis showed that they displayed an unusual nucleotide profile (Fig. 3a and b), confirming that they were not a product of mRNA 3’UTR sequencing. Globally, approximately 2.5% of the alignments were discarded according to the previous reasoning. Alignments that were not discarded during the previous process were further analyzed, and found to mainly align to annotated 3′UTRs regions of the genome (Fig. 3c), supporting the idea that these were in fact genuine sequencing from the 3′end of mRNA transcripts. On the other hand, the discarded alignments usually derived from non-annotated genomic regions, possibly related with transposable elements or other highly variable nucleotide sequences.

Taking all wild-type data sets, 14,593 PASs were reliably assigned to the genomic features listed in the Ensembl Fungi gene annotation for *M. oryzae* version 27 (http://fungi.ensembl.org/), which contains 13,218 annotated features. Each read was assigned to its overlapping feature (including protein-coding genes and the available ncRNAs), ambiguous cases were assigned to the closest 3’ terminal end. To account for inaccurate (usually short) 3’UTR annotations, which are common even in well-annotated genomes^14^, we extended the gene annotation by 400 bp. The selection of this supplemental UTR range was justified by the fact that on average reads were at most 400 bp far from the closest annotated stop codon (Fig. 3d). Reads that could not be assigned to any known feature are not included in this dataset. A PAS was considered to be called with high-confidence if it was detected in at least two of the three replicates according to the following rule: its expression is considered distinct from basal noise if its standard score calculated against the whole gene expression (summing up all PASs expression from that gene) has a confidence level >99% (z-score > 2.58 or z-score < 2.58). The standard score was calculated as: z-score = (value-mean)/std, where “value” is the number of supporting reads for the PAS, “mean” is the mean value of supporting reads for all the PASs in the gene, and “std” is the standard deviation of supporting reads for all the PASs in the gene. PASs with less than five supporting reads in every replicate were discarded. High-confidence PASs that fell within 33 bp of one another (roughly the span of the PAS regulatory region, including the A-rich and the U-rich regions upstream of the cleavage site) were resolved to a single PAS - that being, the one that was most highly expressed in that 33 bp region.

### Code availability

A Galaxy workflow describing the procedure followed to reproduce the PAS identification is available at (Magnaporthe oryzae polyadenylation sites for wild-type and Δrbp35 mutant, Data Citation 1), together with additional scripts required to run the workflow together with a summary of the pipeline.

## Data Records

All sequencing data have been uploaded to the National Center for Biotechnology Information (NCBI) (Alternative polyadenylation controls pathogenicity-associated mechanisms in the rice blast fungus, Data Citation 2), with an overview of the submission provided in Table 1. These data contain paired-end read sequencing for the 24 samples considered: wild-type and Δ*rbp35* mutant strains in four different growth conditions (complete medium (CM), minimal medium (MM), nitrogen starvation (MM-N) and carbon starvation (MM-C)), in three replicates. Each archive is stored in FastQ file format.

Illumina deep sequencing read alignments are provided as BAM files (samtools.github.io/hts-specs/SAMv1.pdf), which are sorted and come with the relative index files (Magnaporthe oryzae polyadenylation sites for wild-type and Δrbp35 mutant, Data Citation 1); Bed Graph files were created in order to ease the visualization of PAS-usage levels and are available in the same deposit, together with Generic Feature Format (GFF) files that represent the high-confidence PASs called by our algorithm. All these files are produced from the final filtered alignment, and can be loaded and viewed with a genome browser like IGV^15^, using version 21 of Ensembl Fungi DNA data and genome annotation (http://fungi.ensembl.org/). The GFF files were used to create the FAIR Linked Data^16^ that was published in Dydra (https://dydra.com) under a Creative Commons license to enable mechanized query of the results (see Usage Notes below). Data files are stored on Zenodo (Magnaporthe oryzae polyadenylation sites for wild-type and Δrbp35 mutant, Data Citation 1).

## Technical Validation

**Figure 4:**
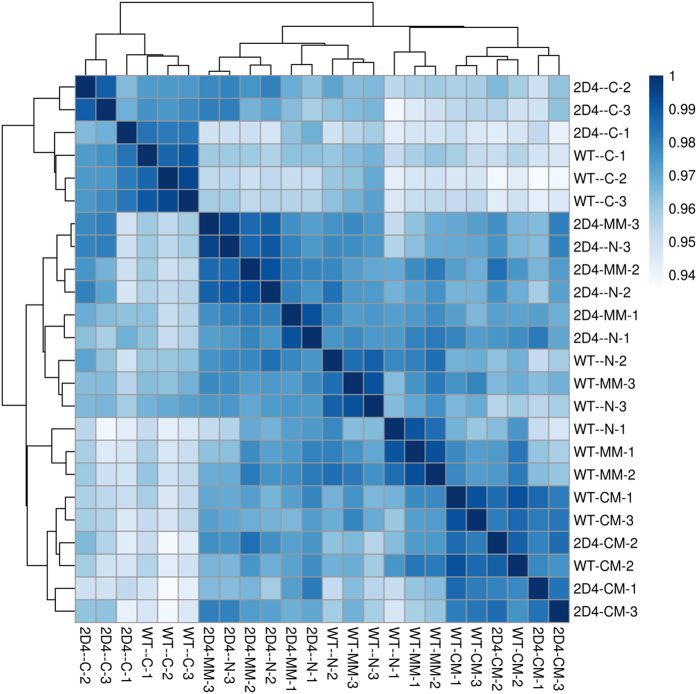
RNA sequencing between-samples correlation diagnostics. Heat map representing the sequencing correlations between the samples. Correlations were calculated using the Pearson correlation coefficient from the log-transformed raw reads counting per gene.

Illumina deep sequencing produced 24 samples providing between between 4,751,592 and 11,517,077 reads per sample (Table 1). FastQC analysis indicated that these reads were generally of high quality (Fig. 2). The correlation between biological replicates, based on calculated gene expression (i.e. number of reads observed for each gene), ranged from 92% to 97%. Correlation matrix analysis revealed a strong similarity between the biological replicates (Fig. 4).

Approximately 73% of reads could be aligned to the *M. oryzae* genome, and about 36% were finally assigned to a high-confidence PAS (as described in methods). Paired-end reads were 44 bp long with a mean mapping quality of 36. Only ∼1% and ∼4% of reads contained illumina adapters or poly(A) tail residues, respectively.

Internal priming was detected and filtered out from the sequencing data using the algorithm described in the methods section, where about 2.5% of the total reads were found to result from internal priming.

As described in methods, reads were assigned to genomic features after an additional 400 bp was included beyond the annotated 3’UTR to compensate for incorrect genome annotations. We justify this by noting that mapped reads are commonly found to accumulate as far as 400 bp from protein-coding genes' stop codons. The typical upstream A-rich region which contains the polyadenylation signal, was found within ∼3 bp from the cleavage site. Consequently, high-confidence PAS detection included the clustering of PASs closer than 33 bp.

## Usage Notes

The publicly accessible triplestore (https://dydra.com/markw/polyadenylation-sites-in-magnaporthe-oryza/) provides a sample SPARQL query representing the query we anticipate to be most informative (i.e. for a given locus, what PASs are used under which conditions). A Web interface has been constructed to enable visualizing the same query results via a Web browser (http://linkeddata.systems/Magnaporthe/polyA_Sites/). Consistent with the FAIR Principles, machine-readable metadata about this SPARQL endpoint is available in VOiD format by resolving the URL of the endpoint, and machine-readable metadata about the data within the endpoint is available by resolving the named graph URI of the dataset. A track hub was also registered, compatible with genome browsers such as Ensembl^17^, under the name ID “Magnaporthe oryzae poly(A) sites” (Magnaporthe oryzae poly(A) sites, Data Citation 3).

## Additional information

**How to cite this article**: Marconi, M. *et al*. Genome-wide polyadenylation site mapping datasets in the rice blast fungus *Magnaporthe oryzae*. *Sci. Data*. 5:180271 doi: 10.1038/sdata.2018.271 (2018).

**Publisher’s note**: Springer Nature remains neutral with regard to jurisdictional claims in published maps and institutional affiliations.

## Supplementary Material



## Figures and Tables

**Table 1:** Statistics and reference information for the data deposits.

Sample	NCBI accession	TOTAL READS	COVERAGE (reads x poly(A))	MAPPED READS	% MAPPED READS	READS TO FINAL POLY(A)	% READS TO FINAL POLY(A)
2D4-C-1	SRR6289253	9159697	416	6910435	75.44%	2658457	38.47%
2D4-C-2	SRR6289252	9109610	414	6435511	70.65%	2161136	33.58%
2D4-C-3	SRR6289255	10024565	456	8129792	81.10%	2981767	36.68%
2D4-CM-1	SRR6289254	8967225	408	7007908	78.15%	2757831	39.35%
2D4-CM-2	SRR6289257	8363408	380	6267714	74.94%	2340051	37.33%
2D4-CM-3	SRR6289256	9445053	429	7021325	74.34%	2635233	37.53%
2D4-MM-1	SRR6289259	8627786	392	6896138	79.93%	2639284	38.27%
2D4-MM-2	SRR6289258	9601345	436	7878426	82.06%	3059830	38.84%
2D4-MM-3	SRR6289251	10833928	492	7667094	70.77%	2801519	36.54%
2D4-N-1	SRR6289250	7179634	326	5832279	81.23%	2265343	38.84%
2D4-N-2	SRR6289243	7914657	360	5570410	70.38%	1879540	33.74%
2D4-N-3	SRR6289242	10194680	463	7615407	74.70%	2800059	36.77%
WT-C-1	SRR6289241	4751592	216	3285309	69.14%	1158885	35.27%
WT-C-2	SRR6289240	9394688	427	6526841	69.47%	2305152	35.32%
WT-C-3	SRR6289247	11517077	524	7150626	62.09%	2587146	36.18%
WT-CM-1	SRR6289246	8650470	393	6739845	77.91%	2599211	38.56%
WT-CM-2	SRR6289245	7586470	345	5858393	77.22%	2305566	39.35%
WT-CM-3	SRR6289244	8010277	364	5241813	65.44%	1790828	34.16%
WT-MM-1	SRR6289249	8486808	386	6088560	71.74%	2219527	36.45%
WT-MM-2	SRR6289248	6046667	275	4595605	76.00%	1718926	37.40%
WT-MM-3	SRR6289238	10121584	460	7302607	72.15%	2526923	34.60%
WT-N-1	SRR6289239	9020001	410	6605602	73.23%	2458719	37.22%
WT-N-2	SRR6289236	9308411	423	6475055	69.56%	2221447	34.31%
WT-N-3	SRR6289237	9777133	444	6920956	70.79%	2520589	36.42%
		8837199	402		73.68%		36.72%
This table provides general quality statistics for the datasets, as well as general metadata related to the individual data deposits.							

## References

[d1] Zenodo Marconi M. Rodriguez-Romero J. Sesma A. Wilkinson M. D. 2018 https://doi.org/10.5281/zenodo.1168454

[d2] Sequence Read Archive 2018 SRP124953

[d3] The Track Hub Registry Marconi M. Rodriguez-Romero J. Sesma A. Wilkinson M. D. 2018 http://trackhubregistry.org/search/view_trackhub/AWGUnQiPPEEPJhVh5mdH 10.1038/sdata.2018.271 PMC6257040 30480660

